# Sleep Enforces the Temporal Order in Memory

**DOI:** 10.1371/journal.pone.0000376

**Published:** 2007-04-18

**Authors:** Spyridon Drosopoulos, Eike Windau, Ullrich Wagner, Jan Born

**Affiliations:** Department of Neuroendocrinology, University of Lübeck, Lübeck, Germany; Claremont Graduate University, United States of America

## Abstract

**Background:**

Temporal sequence represents the main principle underlying episodic memory. The storage of temporal sequence information is thought to involve hippocampus-dependent memory systems, preserving temporal structure possibly via chaining of sequence elements in heteroassociative networks. Converging evidence indicates that sleep enhances the consolidation of recently acquired representations in the hippocampus-dependent declarative memory system. Yet, it is unknown if this consolidation process comprises strengthening of the temporal sequence structure of the representation as well, or is restricted to sequence elements independent of their temporal order. To address this issue we tested the influence of sleep on the strength of forward and backward associations in word-triplets.

**Methodology/Principal Findings:**

Subjects learned a list of 32 triplets of unrelated words, presented successively (A-B-C) in the center of a screen, and either slept normally or stayed awake in the subsequent night. After two days, retrieval was assessed for the triplets sequentially either in a forward direction (cueing with A and B and asking for B and C, respectively) or in a backward direction (cueing with C and B and asking for B and A, respectively). Memory was better for forward than backward associations (p<0.01). Sleep did not affect backward associations, but enhanced forward associations, specifically for the first (AB) transitions (p<0.01), which were generally more difficult to retrieve than the second transitions.

**Conclusions/Significance:**

Our data demonstrate that consolidation during sleep strengthens the original temporal sequence structure in memory, presumably as a result of a replay of new representations during sleep in forward direction. Our finding suggests that the temporally directed replay of memory during sleep, apart from strengthening those traces, could be the key mechanism that explains how temporal order is integrated and maintained in the trace of an episodic memory.

## Introduction

A basic principle in the organization of experience is its development over time. Episodic memory can be seen as a temporal sequence of events that is stored as a declarative memory in a brain system which essentially relies on the function of the hippocampus and closely interconnected regions [1]. An involvement of the hippocampus in memory of temporal sequences has been demonstrated in animals and humans [1], [2]. Rats with hippocampal lesions are unable to learn the order of successively presented pairs of odors [3]. Likewise, humans with damage to the hippocampus are distinctly impaired in explicitly memorizing a sequence of stimulus locations in a serial reaction time task [4], [5]. Although the exact mechanism of how events are stored in time in hippocampal circuits is not yet fully understood, quite elaborated models have been proposed [6]–[10]. Specifically, it was suggested that encoding of event sequences is based on an interaction of autoassociative networks in the CA3 region of the hippocampus, which stabilize the representation of each sequence element, with heteroassociative feedback circuits to the dentate gyrus that enable the chaining of succeeding sequence events [6]. During wakefulness, the encoding of temporal sequences is coupled to the hippocampal theta rhythm and to the phase related coordinate firing of neurons encoding sequential elements with reference to the theta cycle (i.e., phase precession) [11].

There is a rapidly growing body of evidence showing that sleep facilitates the consolidation and long-term storage of new memories, including hippocampus-dependent declarative memories [12]–[15]. In humans, the retention of word-pairs, episodic and spatial memories is distinctly facilitated when learning is followed by a period of sleep in comparison with learning followed by a wake period [16]–[21]. Consolidation of declarative memory was found to profit particularly from retention sleep periods rich in slow wave sleep (SWS), whereas rapid eye movement (REM) sleep is less effective in enhancing such memories [15], [21]. The neuronal mechanisms underlying the consolidation of declarative memories during sleep are less clear. A central assumption is that the consolidation of newly established memories relies on a covert reactivation of those neuronal networks that were active and used for encoding of the events at learning during wakefulness [22]–[27]. It was found that during SWS the firing pattern of hippocampal CA1 cells followed the same temporal order as during prior learning, taking place while the animal was awake [23], [25], [28], [29]. The hippocampal replay which is accompanied by sharp wave-ripple activity and driven repetitively by slow oscillation potentials originating from neocortical networks, is supposed to stimulate a gradual transfer of memory representations for long-term storage into neocortical circuitries [15], [30]–[33]. However, it is an unresolved issue whether the temporally coordinated replay of sequence firing patterns in the hippocampus is indeed critical to episodic memory consolidation. As a matter of fact, it is presently unknown to what extent the sleep-associated consolidation process specifically strengthens the temporal structure underlying a sequence. Alternatively, sleep-associated consolidation of declarative memories could strengthen associations independently of their temporal sequence context. In this view, the sleep-dependent improvement in memory could reflect a more extended representation of the memories within neocortical networks, but the process of consolidation would not require a replay in any temporal order. There is evidence for a backward replay of hippocampal memories although this appears to prevail in the wake state during brief rest periods at learning [34].

Here we addressed the question whether the consolidation of hippocampus-dependent declarative memories during sleep involves specifically the strengthening of the temporal order in a sequence of events. For this purpose, we adopted a design that was previously successfully used to investigate asymmetry in the recall of stimulus sequences probed either in forward or backward direction [35]. Using triplets of words (A-B-C), these experiments confirmed an advantage of forward over backward recall, i.e., a better recall of B in response to the cue word A than in response to the cue word C. The advantage of forward probed recall was subsequently confirmed with longer lists. However, those studies used only short delays between encoding and recall of a few minutes, and did not consider sleep as a factor influencing the consolidation of sequenced associations. In the present experiment, subjects were asked to learn triplets of words before a period of retention sleep (vs. a control period of wakefulness) and recall was tested two days later, probed either in a forward or backward manner. If sleep-associated consolidation strengthens the temporal sequence characteristics within the triplet episode, then the sleep-dependent improvement in recall should be particularly pronounced with forward cueing. However, if sleep strengthens the association between the elements of the triplet independent of its temporal direction, an equal enhancement of forward and backward retrieval should be observed after sleep. Previous studies have shown that the improvement in memory is greater for weakly than for strongly encoded associations in both the declarative and the procedural memory system [36], [37]. At immediate recall testing of triplets, memory for backward associations is generally inferior to the memory for the forward associations, indicating a difference in the encoding strength between the forward and backward associations [35]. Thus, it could be even expected that the benefit from sleep for backward associations is greater than for forward associations, if the sleep associated consolidation process spares the temporal sequence characteristics of the trace.

## Results

**Figure 1:**
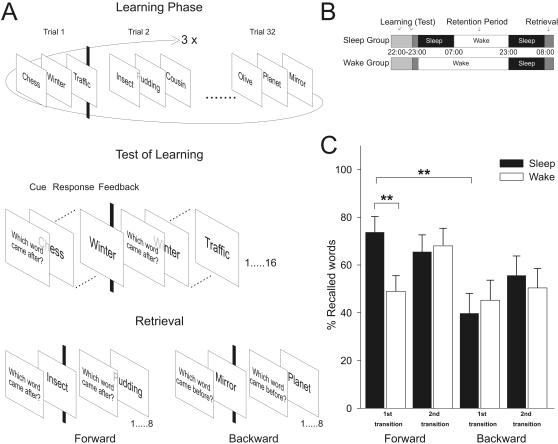
Task description, study design and Retrieval performance. **(A)**Task description and **(B)** study design. Subjects learned word-triplets **(A-B-C)** in the evening starting at 22:00 h. After three successive presentations of all 32 triplets, learning performance (Test of Learning) was determined as a baseline measure for half of the triplets in forward direction. Afterwards the sleep group went to bed whereas the wake group remained awake throughout the night and the following day. In the night afterwards both groups slept normally and returned to the laboratory in the following morning at 8:00 h for retrieval testing. Probed recall testing was performed either in forward direction or in backward direction. **(C)** Retrieval performance. Percentages of retrieved words with reference to baseline determined at the Test of Learning in the sleep (n = 13) and wake (n = 13) group for forward and backward retrieval (for items not previously used at Test of Learning). Subjects in the sleep group were distinctly better in the retention of the forward associations than the subjects in the wake group specifically for the 1^st^ transition. Note also the pronounced improvement in the sleep group in forward associations compared with backward associations again especially for the 1^st^ transition. ** *p*<0.01, two-tailed pairwise comparisons.

For an illustration of the task and study design refer to Figure 1. At learning (before nocturnal retention intervals of sleep and wakefulness) 32 triplets of unrelated words were presented three times, and then baseline learning was determined by assessing memory for half of the triplets, and only in forward direction. At retrieval testing (that took place after subjects had slept regularly on a second night), memory for the other half of triplets was tested. In half of these 16 triplets memory was probed in a forward direction, starting with the first word ‘A’ and requiring the subject to type in the following B word (referred to as 1^st^ or AB transition), and then presenting the second word ‘B’ and asking for the C word (referred to as 2^nd^ or BC transition). For the other half of the 16 triplets, recall was probed in backward direction by starting with the last C word, and asking for the B word (referred to as 1^st^ or CB transition), and then the second B word was presented and the first A word was asked for (referred to as 2^nd^ or BA transition). Retention was determined by calculating the percentage of words for each transition that a subject correctly retrieved at recall testing compared to his/her score at the test of learning. Since at learning only forward recall was probed, percentages were calculated for both forward and backward retrieval with reference to the respective 1^st^ and 2^nd^ transitions of forward recall at learning. Additionally, we assessed recall performance at retrieval testing without reference to learning performance (summarized in Table 1). However, we restrict this report to percent values of recall with reference to learning, since both measures yielded essentially the same results.

**Table 1:** Performance at Learning and Retrieval

	Sleep		Wake	
	1^st^ transition	2^nd^ transition	1^st^ transition	2^nd^ transition
*Test of Learning (% recalled words)*				
Recall (baseline performance)	80.77±6.07	89.42±4.38	76.44±3.82	87.02±4.15
*Retrieval (% recalled words with reference to test of learning )*				
Forward retrieval	73.76±5.41†§	65.56±6.31	48.99±7.52	68.30±7.76
Backward retrieval	39.79±7.88	55.65±9.18	45.27±8.80	50.40±6.91
*Retrieval (% recalled words – unreferenced to test of learning)*				
Forward retrieval	58.65±5.55†§	59.62±6.66	39.42±6.46	61.54±7.81
Backward retrieval	35.58±6.91	51.92±8.82	37.50±7.75	46.15±7.41

Learning performance (top, Test of Learning), retrieval performance with reference to test of learning (middle), and retrieval performance unreferenced to test of learning (bottom). At the test of learning (recall performance with reference to 16 triplets tested) there were no significant differences between any conditions, except that recall for the 2^nd^ transitions was generally superior to recall for the 1^st^ transitions. Retrieval performance was determined for the triplets that were not used at the test of learning, and is indicated separately for 1^st^ and 2^nd^ transitions and forward and backward testing. The middle panel indicates retrieval performance as percentage of recalled words with reference to the number of recalled words at the test of learning. The bottom panel indicates retrieval performance with reference to the 8 word-triplets per testing category (forward, backward). (Percent scores of retrieval performance were calculated for each individual subject). Note, compared with wakefulness, retrieval after sleep is particularly enhanced for 1^st^ transitions tested in forward direction († p = 0.01). Retrieval for the 1^st^ transition after sleep is also significantly better in forward direction compared to backward direction (§ p<0.01). Data are mean±SEM.

### Analysis of retrieval

The main finding of this study is that subjects in the sleep group were better in the retention of the forward associations than the subjects in the wake group, which was specifically evident for the 1^st^, i.e. AB transitions (Figure 1). Data from retrieval testing were analyzed in a 2×2×2 repeated measurements analysis of variance (ANOVA) with the between subjects factor sleep/wake and the within subjects factors forward/backward retrieval and 1^st^/2^nd^ transition in the memory test. For mean±SEM percentages of retrieved words refer to Table 1. The analysis revealed that memory retention was generally better for the words retrieved in forward than backward direction [*F*(1,24) = 12.26, *p* = 0.002] and also for the words in the 2^nd^ as compared to the 1^st^ transition [*F*(1,24) = 5.03, *p* = 0.034]. Yet, the main finding showed that sleep enhanced retention over wakefulness specifically in forward retrieval for the 1^st^ transition [significant three-way interaction *F*(1,24) = 11.78, *p* = 0.002, Figure 1]. This effect was further confirmed in subsequent post-hoc analyses by pairwise comparisons, showing that the sleep and wake groups only differed in the percentage of retrieved words in forward retrieval for the 1^st^ transition (*p* = 0.01). Additionally, with respect to differences *within* the groups, the sleep group was distinctly better in the forward as compared to the backward association (*p* = 0.007), but this effect was stronger for words in the 1^st^ than 2^nd^ transition (*p* = 0.008). The wake group on the other hand showed better retention for words in the 2^nd^ transition irrespective of direction (*p* = 0.007). Here, forward retrieval was slightly better compared to the backward direction of retrieval but this effect was not significant (*p* = 0.12), nor was the interaction between direction and transition significant in this group (*p* = 0.11).

### Performance at learning

Data from immediate recall testing at learning before the retention intervals of sleep and wakefulness were analyzed in a 2×2 repeated measurements ANOVA with the between subjects factor sleep/wake and the within subjects factor 1^st^/2^nd^ transition in the memory test. The analysis did not reveal any differences between the groups (*p*>0.60, for all comparisons, see Table 1), this way assuring that the groups were fully comparable at encoding. Only the factor transition was significant showing that more words in the 2^nd^ transition were retrieved as compared to the 1^st^ [*F*(1,24) = 19.47, *p*<0.001].

### Control of general retrieval capability and self-ratings

As an estimate of the subject's general capability to retrieve information from long term memory we used a “word fluency task” [38] where subjects had to generate and write down as many words starting with a certain letter (“M” or “P”) as they could within two minutes. Performance on this task did not differ between sleep and wake conditions neither before learning (21.07±1.16 and 19.85±0.93, respectively) nor before retrieval testing (22.07±1.10 and 19.40±1.29; *p*>0.16 for all comparisons).

Subjects of the sleep and wake conditions also did not differ with respect to the rating of sleep quality (on a five-point scale, with “1” representing poor sleep) in the second night before retrieval testing. Rated sleep quality was practically identical in both groups (both 3.83±0.21, *p*>0.99). Subjective ratings of concentration, tiredness, and mood after the learning phase and after retrieval testing also did not indicate any differences between sleep and wake conditions (*p*>0.13) except that compared with the sleep group, subjects of the wake group reported to feel less tired at learning (2.62±0.18 versus 3.61±0.27) and more tired after retrieval testing (4.00±0.28 versus 2.15±0.22, *p*<0.01, for both comparisons). While the difference at learning reflects most likely the anticipation of the forthcoming periods of sleep versus wakefulness, the difference at retrieval could hint at persisting sleep inertia after recovery sleep. To assure that such influences did not affect memory retention, we re-analyzed retrieval performance as well as immediate recall performance at learning using rated tiredness as covariate in the original 2×2×2 repeated measures ANOVA. The factor was not found significant alone or in interaction with any of the other variables (*p*>0.26 for all analyses). Moreover, these analyses confirmed (*p*<0.05) all effects of the original analyses reported above, including that of sleep versus wakefulness on retention for the 1^st^ transition in forward direction.

### Polysomnographical recordings and cortisol concentrations

Polysomnographical data scored off-line according to standard criteria [39] showed that subjects in the sleep condition slept normally in the laboratory on the night following the learning phase. Sleep time amounted to (mean±SEM) 451.17±5.79 min. The percentages of sleep time in wakefulness, sleep stage 1 (S1), sleep stage 2 (S2), SWS and REM sleep were respectively: 1.61±0.93, 3.71±0.57, 46.19±1.65, 25.37±1.66, 21.53±1.27%.

Cortisol concentrations were measured in saliva against the background of previous studies indicating substantial influences of corticosteroids on memory retention and retrieval [40]–[42]. Cortisol concentrations did not differ between the sleep and wake groups at learning (collapsed across values before and after learning in µg/dl, sleep: 0.05±0.01, wake: 0.08±0.02) or at retrieval testing (sleep: 0.57±0.09, wake: 0.56±0.15, (*p*>0.83 for all comparisons). Also, the groups did not differ significantly in cortisol concentration in the morning after the night at the laboratory (sleep: 0.47±0.05, wake: 0.41±0.09, *p*>0.60). Due to the circadian rhythm, cortisol concentrations were generally higher in the morning at recall testing than in the evening during the learning phase (*p*<0.001).

## Discussion

In this study we tested whether sleep-dependent memory consolidation affects the associative strength of forward and backward associations of a temporal sequence. Our data indicate that sleep after learning, in comparison to wakefulness, enhances memory for the forward associations of learned word-triplets. This effect concentrated on the 1^st^, i.e. the AB transition. There was no effect of sleep on backward retrieval of the triplets which, as expected, was generally inferior to forward retrieval. This outcome supports the notion that the consolidation of hippocampus-dependent memories during sleep comprises the strengthening of the underlying temporal structure in a sequence of events.

The subjects' performance in the sleep and wake groups was comparable at encoding. Also subjects had to achieve a score of at least 50% correct responses at baseline learning. Hence, the possibility that differences in the strength of encoding, shown in previous studies to modify the need for consolidation [37], can be ruled out as an explanation for the obtained effect of sleep on retention performance. Moreover, cognitive performance on a word fluency task showed that the groups were fully comparable with respect to general capabilities to retrieve information from memory. Circadian variations that could differentially affect memory performance can also be excluded as sleep and wake groups were tested at the same time points during the circadian cycle. Subjective ratings of mood and feelings of activation likewise indicated comparability of the two conditions, except that subjects in the wake group felt less tired at learning but more tired after retrieval testing than those of the sleep group. However, additional analyses revealed that this difference in rated tiredness remained without effect on recall performance. Moreover, concentrations of the stress hormone cortisol known to affect memory function [41]–[43] did not differ between the sleep and wake groups during encoding or retrieval. In light of these controls, the effect of sleep on retention cannot be explained in terms of unspecific influences on cognitive function. Most importantly, any confounding influence of an acute sleep deficit on retrieval performance was avoided here by testing retrieval not until a second night had passed, which allowed for recovery sleep in the wake condition. On the other hand, the effects of post-learning sleep specifically on memory retention of forward associations might have been even more pronounced without this delay, because sleep in the second night after learning could have contributed to processes of sleep-dependent consolidation to some degree also in the wake condition, thereby diminishing the difference in retention performance between sleep and wake conditions.

The finding that forward retrieval was generally better than backward retrieval confirms previous findings on asymmetry for serial lists [35], [44]–[46]. Notably, in some of these previous studies the words were not only presented in a temporal order but also in different spatial locations. Since additional spatial information can distinctly improve backward recall [45], in our study the words were presented successively in the middle of the computer screen to prevent any spatial confound. This lack of spatial information might explain why our subjects achieved relatively lower accuracy rates with backward probing in comparison to other studies [e.g. ref. 35].

Although the backward associations between the words are weaker than the forward associations, sleep benefited selectively the consolidation of forward associations. This finding contrasts with forgoing findings indicating a greater memory benefit from sleep of associations that were less intensely encoded before sleep due to a lower learning criterion or an interfering task [37]. In combination with that data, the absence of any sleep-dependent enhancement for the backward transitions supports the notion that forward and backward recall rely on different memory processes [35], [44]–[46]. In fact, in showing that post-learning sleep specifically strengthens forward associations in memory, our findings essentially expand this notion by indicating that the forward/backward asymmetry in sequential memory becomes embedded in the trace in the course of consolidation, independent of retrieval strategies that can add to temporal sequence structuring [47]. Thus, our finding suggests that the temporally directed replay of memory during sleep, apart from strengthening those traces, could be the key mechanism that explains how temporal order is integrated and maintained in the trace of an episodic memory [6], [23].

We deliberately only used forward testing at the test of learning before the retention intervals because backward testing at this stage would induce a number of cognitive processes interfering with the natural forward encoding of episodes in regular temporal order. The encoding process in the study was to be kept as normal as possible. Importantly, a backward testing in the immediate aftermath of presenting the words would stimulate a general tendency in the subject to bring the triplets into mind in reversed (i.e., backward) order, thereby covertly reversing the natural forward direction as basal reference of encoding. Consequently, backward testing during the learning phase was abandoned to reduce such confounds of temporal direction to a minimum. Still, it could be argued that forward testing of the words at the test of learning to a certain degree induced some anticipatory effects in favor of forward direction at memory testing after the retention interval. However, this effect supposedly affected primarily the items that were used at the test of learning since only those items were explicitly tested in a forward direction. Because these words were not used for determining retrieval performance, the possibility of such a contamination in the present data set is greatly diminished. Of course, subjects would likely anticipate a forward test rather than a backward test at retrieval even if the forward testing at baseline were omitted, and effects of sleep might be just a matter of consistency in temporal structure at initial testing and delayed retrieval testing. In this general form, the forward direction of anticipations deriving from episodic memories is an inherent feature of such anticipations that is difficult to dissociate from the experienced episode per se. Moreover, there is presently little evidence that anticipatory processes, apart from their influence on encoding, could differentially affect consolidation during sleep and wakefulness. Accordingly, additional strengthening of forward associations due to possible effects of test anticipation – whether occurring spontaneously or induced by forward testing at baseline – should contribute only to the general superiority of forward over backward retrieval (i.e. the main effect of testing direction), but not to the sleep-specific enhancement of forward retrieval. As mentioned, several previous studies have shown for different domains of memory that sleep preferably consolidates weak rather than strong associations [36], [37]. Thus, sleep would be even expected here to strengthen backward more than forward associations if direction in temporal sequence per se were not critical to consolidation.

The 2^nd^ transitions were generally better retrieved than the 1^st^ transitions of the word-triplets, both in forward and in backward retrieval which replicates previous studies [35]. This is because for the 2^nd^ transition the subjects have two cues to rely on, which makes the retrieval of these words easier as compared to the 1^st^ transition. Especially since the 2^nd^ transition word was probed immediately after the 1^st^, it is very likely that the availability of two cues significantly decreased the difficulty retrieving the 2^nd^ transition. On this background, our finding that with forward testing of retrieval, sleep specifically enhanced memory for the 1^st^ transition is compatible with previous observations showing that sleep enhances memory for weak associations [37]. In this view, any beneficial effects of sleep-induced consolidation for the 2^nd^ transition was masked by the stronger cueing of this transition. However, since the influence of sleep spared backward associations, associative strength obviously represents a factor secondary to temporal order in declarative memory consolidation during sleep.

It has been argued that signs of reprocessing during sleep merely reflect residual activity in neural assemblies used previously for encoding, whereas the enhancing effect of sleep on memory results from a general downscaling of synaptic efficiency [48]. That view predicts that downscaling erases weak synaptic connections below a certain threshold whereas strong connections survive. This contradicts the present findings of a greater sleep-dependent gain for the weaker rather than stronger associations with forward retrieval. An erasure of weak associations as a result of sleep-associated consolidation is also not supported by our data, since there were no significant differences between sleep and wake conditions in the recall of the backward associations being distinctly weaker than the forward ones. Indeed, the outcome of our study speaks for the notion that consolidation during sleep involves active replay of memory traces, likely occurring within a dialogue between hippocampus and neocortex [15], [27], [30], [49]. Importantly, the direction of replay is of particular relevance in this view since it determines the temporal order in the sequence of consolidated events. This is reminiscent of ideas by Waugh [47] who viewed the asymmetry in retrieval direction of serial lists as an epiphenomenon resulting from rehearsal of the sequences. On the other hand, our data suggest that backward memory replay as observed in waking animals during spatial exploration [34] should not contribute to consolidation of sequence and episodic memories during sleep. Along this line of reasoning, our results agree with concepts of sequence memory pinpointing that sequence structure is preserved during replay in the sleep state although it can occur at a faster rate than during waking [6], [23], [28], [29]. Thus, the temporally ordered replay of newly encoded memories during sleep, taking place in interconnected autoassociative and heteroassociative networks of CA3 and dentate gyrus in the hippocampus might well provide a mechanism that enhances the sequence structure of episodic memories during the process of consolidation.

## Materials and Methods

### Subjects

Twenty-eight healthy, non-smoking, drug free, native German speaking subjects with no prior history of sleep disturbances and with regular sleep-wake cycle participated in the experiments and received a money reward for their participation (14 males, 14 female, mean age 24 years, range 19–34 years). Data from two women were excluded from analysis: one from the sleep group who had not reached the cut-off score of 50% correct response words at learning and one from the wake group who did not comply with the instruction to remain awake during the day. The experiments were approved by the local ethics committee. All participants gave written informed consent before participation. All participants were tested individually. Prior to the experiments proper, subjects in the sleep condition had an adaptation night in the laboratory including the placement of electrodes. The participants were instructed to get up at 7:00 h and not to take any naps on the days of the experiment. They also had to abstain from taking any caffeine containing drinks at least 6 hours before learning and retrieval sessions. Alcohol was not permitted on the experimental days in any condition and throughout the whole experimental period.

### Procedure and Design

In the sleep condition, subjects reported to the laboratory around 21:30 h. Following the placement of electrodes and preparations for bedtime, the learning phase started at 22:00 h. To assess general capability for retrieving information from memory, subjects first performed the word fluency task [38]. They were provided with a sheet of paper where they had to write down within 2 min as many words as they could, starting either with the letter M or P, the order of which was balanced across subjects. Thereafter, subjects performed on the triplets-memory task. They were seated in a room in front of a computer screen where the 32 word-triplets of the task were presented after they had been instructed to memorize the three words of each triple as belonging together (see Figure 1). The set of 32 triplets was presented successively 3 times (in different order) and then memory was probed for half, i.e. 16 of the triplets in order to estimate baseline learning. Learning and baseline testing took approximately 40 min. Subjects were then instructed not to rehearse the words any more. In the end of the learning phase, subjects completed a self-rating questionnaire consisting of 5-point rating scales concerning feelings of activation, tiredness, motivation, attention, concentration and other psychological and physiological characteristics that might exert unspecific effects on cognitive functioning. They were then offered to go to the toilet, after which they immediately went to bed. Lights were turned off within 10 min after learning was completed. At 07:00 h the next morning subjects were awakened, and electrodes were removed. Before leaving the laboratory, subjects were told that they should follow their regular activities during this day, to go to bed around 23:00 h, and not to rehearse the triplets. Subjects reported back to the laboratory the next morning at 08:00 h for retrieval testing. Caffeine containing drinks were again not allowed on this morning. Before retrieval testing the word fluency task was administered again. After having performed the retrieval test on the triplets-memory task, the participants completed again the self-rating questionnaire as they had after the learning phase. Additionally, they rated on a 5-point scale the quality of their sleep the night before. At the end of the session, by a standardized interview it was assured that the subjects had fully complied with the instructions, and subjects also had to report the activities they had engaged in the day before. Also the subjects were explicitly asked in post-experimental questionnaires to indicate whether they had intentionally or unintentionally rehearsed the words during the retention period. (Note that even if some rehearsal remained undetected, this would be expected to act towards weakening the presumed memory enhancing effect of sleep, because the probability of rehearsal is higher during the wake condition.)

Since glucocorticoids are known to influence memory function [40]–[42] saliva cortisol was sampled, as a measure of adrenocortical secretory activity, both before and after learning and retrieval, as well as before leaving the laboratory after the first night.

In the wake condition, the procedure was the same except that after learning was completed subjects remained awake throughout the night and the following day. They stayed in the laboratory under the supervision of an experimenter until 07:00 h and afterwards went home. They were instructed not to go to bed before 23:00 h on this day. Throughout the experiment they wore an “activity watch” (Actiwatch® system, Cambridge Neurotechnology Ltd., Cambridge, UK), which was used to assure that subjects had no brief minute periods of sleep during the experimental interval of wakefulness. During the night, they were engaged in standard activities, including watching movies, playing computer games, and going out for a walk in the experimenter's company. Reading was not allowed.

### Memory Task and Materials

The words were taken from lists used in previous experiments in our laboratory (see Supporting Information “Materials S1”, for the lists and a translation into English). Words in the lists were semantically unrelated. They contained 6–8 letters and had no more than three syllables. Half, i.e. 16, of the 32 triplets was used for determining baseline learning performance before the retention period (Test of Learning; Figure 1A). The other half was used in the retrieval test after the retention interval. Triplets used for determining learning and retrieval testing were balanced across subjects. At retrieval testing, eight of the 16 triplets were used to probe memory in a forward direction whereas the other eight triplets were used for backward memory testing. At learning each word appeared in the middle of the screen for 4 sec, immediately followed by the succeeding word of a triplet. Between the strings of triplets a fixation cross was presented for 4 sec. The list of 32 triplets was presented 3 times in total. Immediately afterwards, baseline learning (test of learning) was assessed on 16 of the triplets. First, for 1.5 sec the question appeared “Which word came after?” The first word of a triplet was presented and remained on the screen for 15 sec during which time the subjects could type the response word (which appeared in the lower left corner of the screen). Feedback was provided irrespectively of the subject's answer by presenting the correct response word for 1.5 sec. The question appeared again for 1.5 sec and then the second word of the same triplet was presented in the same procedure as for the first word, ending again with feedback by presenting the correct response word for the 2^nd^ transition. A fixation cross appeared on the screen for 4 sec indicating the start of a new triplet. At the test of learning, a cut-off criterion of at least 50% correct responses across both triplet transitions was set in order to assure that subjects had indeed followed the instructions and learned sufficiently.

At retrieval testing no feedback was provided. As mentioned above, determination of retrieval performance only referred to triplets *not* used for examination of baseline performance. This is because baseline testing itself entails an additional elaboration of the items tested and an explicit linking of cue words to operations of retrieval in forward direction, which would not only generally enhance memory retention of these items but also obscure differential effects on forward/backward associations. For control purposes, we additionally probed retention of the 16 triplets that were already used for estimating baseline performance, and found that in most subjects recall rate for these items at retrieval was indeed close to 100%, indicating a ceiling effect.

### Data analysis

Retention was determined by calculating the percentage of words for each transition that an individual subject correctly retrieved, with reference to his/her score at baseline learning. Because at learning only forward recall was probed, the calculation of the percentages for backward retrieval was based on the performance for the respective forward transition at the test of learning. This way memory for the AB transition at the test of learning served as baseline for memory of the CB transition at backward testing during retrieval testing, and memory for the BC transition at the test of learning served as baseline for memory of the BA transition at backward testing during retrieval testing.

## Supporting Information

Materials S1 (0.03 MB DOC)
