# Supplementary material for: Sleep Enforces the Temporal Order in Memory
Source: PLoS One. 2007 Apr 18;2(4):e376. doi: 10.1371/journal.pone.0000376 (PMC1849893; doi:10.1371/journal.pone.0000376)
Supplement: Materials S1 — (0.03 MB DOC) [file pone.0000376.s001.doc]

German List

Telefon - Asien - Profil

Vorfall - Bauch - Plakat

Schlamm - Beruf - Besitz

Branche - Blick - Hektar

Zigarre - Brett - Hammer

Merkmal - Keller - Brief

Apparat - Zimmer - Dauer

Spirale - Beweis - Drama

Strasse - Schach - Feuer

Auftrag - Niveau - Figur

Frage - Kirche - Flasche

Fuchs - Inhalt - Amateur

Grund - Winter - Meister

Halle - Klippe - Wirkung

Hotel - Gehirn - Haltung

Hütte - Geruch - Legende

Kreis - Brandy - Angebot

Kugel - Göttin - Konzept

Maler - Redner - Anstand

Markt - Metall - Fahrrad

Schiff - Neigung - Möbel

Vulkan - Wiege - Nagel

Insekt - Pudding - Neffe

Zufall - Spiegel - Pfeil

Becher - Diamant - Rolle

Anteil - Zeitung - Salat

Betrag - Meinung - Seele

Sonate - Wolle - Staub

Detail - Zukunft - Stoss

Anlass - Akustik - Stuhl

Schaum - Klavier - Tinte

Anfang - Gedicht – Vogel

A second version of the list was formed by exchanging the first and third words in each triplet. Both versions were used balanced across subjects in each condition.

English translation of the triplet list

Telephone - Asia - Profile

Incident - Belly - Poster

Mud - Profession - Property

Industry - View - Hectare

Cigar - Board - Hammer

Characteristic - Cellar - Letter

Apparatus - Room - Duration

Spiral - Proof - Drama

Road - Chess - Fire

Order - Level - Figure

Question - Church - Bottle

Fox - Content - Amateur

Reason - Winter - Master

Hall - Cliff - Effect

Hotel - Brain - Attitude

Hut - Smell - Legend

Circle - Brandy - Offer

Ball - Goddess - Concept

Painter - Speaker - Decency

Market - Metal - Bicycle

Ship - Inclination - Furniture

Volcano - Cradle - Nail

Insect - Pudding - Nephew

Coincidence - Mirror - Arrow

Cup - Diamond - Roll

Portion - Newspaper - Salad

Amount - Opinion - Soul

Sonata - Wool - Dust

Detail - Future - Kick

Occasion - Acoustics - Chair

Foam - Piano - Ink

Beginning - Poem - Bird
